# Conservation and Dynamics of Maize Seed Endophytic Bacteria Across Progeny Transmission

**DOI:** 10.3390/microorganisms12122399

**Published:** 2024-11-22

**Authors:** Kaihui Zhai, Yingying Zhang, Caihong Zhao, Qing Wang, Xiquan Gao

**Affiliations:** 1 State Key Laboratory of Crop Genetics & Germplasm Enhancement and Utilization, Nanjing Agricultural University, Nanjing 210095, China; 2022201032@stu.edu.cn (K.Z.); yyzhang@ipsnanjing.cn (Y.Z.); 2021101074@stu.edu.cn (C.Z.); qingwang@njau.edu.cn (Q.W.); 2 Collaborative Innovation Center for Modern Crop Production Co-Sponsored by Province and Ministry (CIC-MCP), Nanjing Agricultural University, Nanjing 210095, China; 3 College of Agriculture, Nanjing Agricultural University, Nanjing 210095, China

**Keywords:** maize, seed, endophytic bacteria, vertical transmission, metabolism

## Abstract

Maize (*Zea mays* L.) is an important cereal crop species for food, feedstock and industrial material. Maize seeds host a suitable ecosystem for endophytic bacteria, facilitating seed germination and seedling growth. However, the inheritance, diversity and potential function of seed endophytic bacteria in maize remain largely unexplored. In this study, the endophytic bacteria in the seeds of maize inbred line WU109 collected during three consecutive seasons were identified using 16S rDNA sequencing. Core community composition was essentially consistent across three seed generations and two planting locations. In total, 212 operational taxonomic units (OTUs) belonging to 11 phyla were identified, among which proteobacteria was the dominant phylum. Fifty-six OTUs were conserved across three seed generations. Within them, 16 OTUs were core components and the dominant OTUs were *Ralstonia solanacearum*, *Delftia tsuruhatensis*, *Bacillu svelezensis* and *Shigella boydii*, accounting for 60% of the total abundance of OTUs. COG and KEGG analyses showed that the function of seed endophytic bacteria was mainly enriched in metabolic processes, especially in amino acid, carbohydrate and energy metabolism. Taken together, the results suggested that the community of maize seed endophytic bacteria was likely co-shaped by both genetic determination and the environment, while the core constitutes of seed endophytes were largely conserved due to transgenerational transmission. Establishing the mutualistic link between the maize seed and its endophytic bacteria enables the exploitation of the potential of endophytes for enhancing crop production. This finding provides a reference to better understand the inheritance and composition of seed core endophytic bacteria in maize.

## 1. Introduction

Endophytic bacteria ubiquitously exist in all terrestrial plants without visible detriment to the host [1]. Various plant tissues host large numbers of endophytic bacteria, which can be recruited either horizontally from external environments or acquired vertically through transgenerational transmission via seeds [2]. Bacterial endophytes preserve a symbiotic relationship with host plants, and they can facilitate the acquisition of nutrients, participate in the regulation of phytohormones to promote plant growth, and release antimicrobial compounds, competing for space and nutrients with phytopathogens to help host plants further mitigate the consequences of harsh conditions [3,4,5]. Endophytic strains affiliated with *Bacillus*, *Enterobacter*, *Burkholderia*, *Pseudomonas*, *Rahnella*, *Azotobacter*, *Serratia*, *Arthrobacter*, *Streptomyces* and *Isoptericola* had been successful when utilized in alleviating abiotic and biotic stress and promoting seedling growth in multiple crop species [6,7,8,9,10,11]. With the rapid development of high-throughput sequencing technology, investigation of the community composition of the microbiome inside plant tissues becomes feasible, providing a better opportunity to understand the mutualistic interactions between host plants and endophytic bacteria [3]. Nevertheless, current researches on plant endophytes mainly focus on the rhizosphere and phyllosphere, whereas the seed endophytes are inadvertently neglected.

The seed phase is an important phase in the crop lifespan, and seeds can be stored for several years when adverse conditions are met, becoming new seedlings in an appropriate environment [12]. Meanwhile, the seed is also an ideal habitat for endophytic bacteria. It has been reported that the dominate phyla of seed bacterial endophytes are *Proteobacteria*, *Actinobacteria* and *Firmicutes*, composed of 80, 25 and 20 genera, respectively, among which the genera *Bacillus* and *Pseudomonas* are ubiquitous [13,14]. The potential applications of seed endophytic bacteria are diverse, involving biofertilization, bioenergy production and bioremediation. *Paenibacillus* strains isolated from wheat seeds could promote seedling establishment and enhance resistance to *Fusarium graminearum* [15], while *Bacillus velezensis* isolated from maize seeds had the ability to promote seedling growth and prevent the infestation of *F. verticillioides* [16]. The inoculation of seeds with *Methylobacterium* sp. Cp3, a core strain of seed endophyte in *Crotalaria pumila*, could promote seed germination, root extension and enhance heavy metal tolerance [17]. Moreover, inoculation with *Pantoea* and *Pseudomonas* strains isolated from rice seeds could enhance plant height and root length [18], whereas elimination of the endophytic bacteria in rice seeds resulted in a remarkable suppression of seedling growth and root hair development [19]. However, it remains unclear where these functional endophytes come from and whether and how the community composition of seed endophytes is changed across seed generations and different ecosystems.

Core bacterial seed endophytes can be transmitted through seed generations, which is known as vertical transmission [20,21,22]. The vertical transmission of core seed endophytic bacteria benefits plant growth at an early stage and confers resistance to phytopathogens. Through an evaluation of two consecutive generations of rice seeds, *Stenotrophomonas maltophilia* and *Mycobacterium abscessus* were found to be conserved in both generations [23]. *Sphingomonas melonis* transmitted across generations in resistant rice seeds conferred resistance to bacterial diseases by generating anthranilic acid [24]. Moreover, *Methylobacterium* sp. Cp3 was found to be the dominant seed endophyte transmitted across seed generations of *Crotalaria pumila*, displaying the potential to promote seed germination and seedling growth [17]. In addition to vertically transmitted seed endophytes, alien seed endophytes transferred by horizontal transmission are also beneficial to host plants through symbiosis [25]. These alien seed endophytes are largely acquired from the environment. A number of studies have comprehensively investigated the community and function of maize seed endophytes, showing their capability to stimulate seedling growth and protect the host from various stresses [16,26,27]. However, details about the composition and conservation of maize seed core endophytic bacteria across consecutive generations remain unclear.

To illustrate the inheritance and potential functions of maize seed bacterial endophytes through diversity, community composition and functional analysis, 16S rDNA sequencing was deployed to identify the community structure of seed endophytic bacteria in three successive seed generations of maize inbred line WU109 in this study. It was found that the community of seed endophytic bacteria was determined by both plant genotype and the environment, and the core community was conserved across transgenerational transmission, which may contribute to the improvement of maize production via the regulation of the metabolism process. Our study elaborated upon the core seed-borne bacteria in maize seeds and provided genetic insights into the function of seed bacterial endophytes, which is helpful for further understanding the complexity of interactions among host plants, endophytes and the environment.

## 2. Materials and Methods

### 2.1. Plant Materials

Maize seeds (inbred line WU109) of three consecutive generations were collected from two planting locations. Seeds from the generations T_0_ and T_1_ were collected from Xinxiang, Henan province, China, located at 35°22′18.48″ N, 113°54′21.53″ E, in 2018 and 2019, respectively. T_2_ seeds were collected from Sanya, Hainan Province, China, located at 18°24′0.18″ N, 109°45′9.00″ E, in 2020. Collected seeds were stored at −80 °C freezer (Zhongkeduling, Hefei, China) for subsequent experiments.

### 2.2. Seed Surface Sterilization

The seeds were sterilized with 1% sodium hypochlorite (NaClO) in a 50 mL sterilized tube for 10 min and stirred with a glass stick constantly, under an aseptic environment and room temperature condition. Following the discarding of NaClO, the seeds were washed thoroughly using sterilized ddH_2_O, then immersed with 75% ethanol for 2 min and washed using sterilized ddH_2_O three times. To confirm the effect of sterilization, a small amount of the final washing water was dropped on the PDA plate, and the immersed water of unsterilized seeds was used as a positive control.

### 2.3. Identification of Seed Endophytic Bacteria by 16S rDNA Sequencing

The sterilized seeds were frozen with liquid nitrogen and thoroughly ground by a plant tissue crusher (ShanghaiJingxin, Shanghai, China). Extraction of total DNA was performed using the MolPure^®^ Soil DNA Kit (18815ES70, YEASEN, Changsha, China) according to the manufacturer’s instructions. Two biological repeats with 10 seeds per replicate were included. The quantity and quality of the extracted DNA were assessed by agarose gel electrophoresis and NanoDrop one (Thermo Scientific China, Shanghai, China). Bacteria-specific primers (799F/1193R) were employed to amplify the V5–V7 region for amplicon sequencing using the Illumina NextSeq 2000 sequencing platform (Illumina China, Shanghai, China). The target sequences were amplified using PCR, and the resulting products were purified, quantified and standardized to generate a sequencing library. Library quality control was conducted to ensure the construction of high-quality libraries.

### 2.4. Data Analysis of 16S rDNA Amplicons

The acquired sequencing data were processed using Qiime 1.9.1. The quality of paired-end reads was checked and demultiplexed using Flash 1.2.11. Uparse 11, RDP classifier 2.13 and Usearch 11 were used to generate OTU information. To obtain reliable OTU data, mitochondrial and chloroplast reads, low-abundance OTUs (less than 0.01%) and that unclassified at the phylum level were removed. The α diversity analysis was conducted using R package vegan 2.6. Principle coordinate analysis (PCoA) for β diversity was conducted by R package scatterplot3d 0.3–44. Population community analysis at the phylum and genus levels was conducted by R package plyr 1.8.9, tidyverse 2.0.0 and ggalluvial 0.12.5. PICRUSt 1.1.0 software based on the COG (Cluster of Orthologous Groups of proteins) and KEGG (Kyoto Encyclopedia of Genes and Genome) databases was used to predict functional gene composition in the samples by comparing species composition information obtained from 16S sequencing data, and then functional differences between samples or groups were analyzed.

## 3. Results

### 3.1. Analysis of Pyrosequencing Data

Surface sterilization is paramount for analyzing endophytic bacteria. After culturing for 3 d, compared to the unsterilized control, no colony was found to be growing on the PDA plate, indicating that the sterilized seeds were suitable for constructing the 16S rDNA library. When the sequencing throughput reached 10 kb, the Shannon index at the OTU level was no longer increased, indicating that the data adequately captured the species diversity in the samples. In total, 257 OTUs and 86,584 reads were acquired. After filtering out the reads from the mitochondria/chloroplast and low-abundance OTUs (less than 0.01%), an average of 10,848 reads affiliated with 212 OTUs were obtained from all the samples.

### 3.2. Community Diversity of Seed Endophytic Bacteria

**Figure 1:**
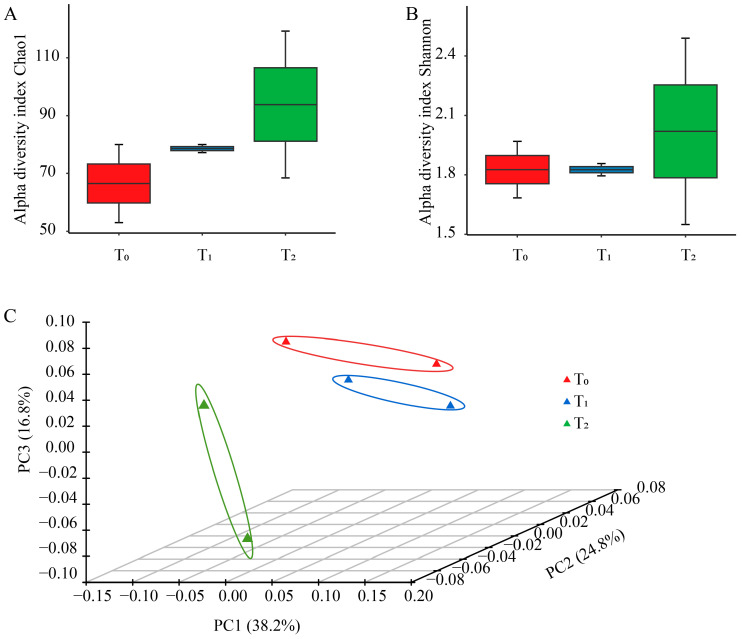
Community diversity for maize seed bacterial endophytes of three seed generations. (**A**) Chao1 richness index of seed endophytic bacteria at OTU level. (**B**) Shannon index of seed endophytic bacteria at OTU level. (**C**) Principal coordinate analysis of total seed endophytic communities across three seed generations at OTU level. T_0_, T_1_ and T_2_ represent three consecutive seed generations, respectively.

The Chao 1 and Shannon index affiliated with alpha diversity were utilized to reflect the richness and diversity of seed bacterial endophytes among the three seed generations. The means of Chao 1 richness calculated for consecutive generations were 66.5, 78.6 and 93.8 in T_0_, T_1_ and T_2_, respectively. Similarly, the mean of the Shannon index of T_2_ and was the highest among the three seed generations, larger than that of T_0_, and T_1_ (Figure 1A,B). For principal coordinate analysis, 79.8% of the variance was explained by the first three coordinates. The distance between T_0_ and T_1_ was short, while T_2_ was far from them (Figure 1C), suggesting that the community structure of T_0_ and T_1_ was more similar than that of T_1_ and T_2_, likely due to the difference between planting areas.

### 3.3. Core Endophytic Bacteria Were Common Among Three Seed Generations

**Figure 2:**
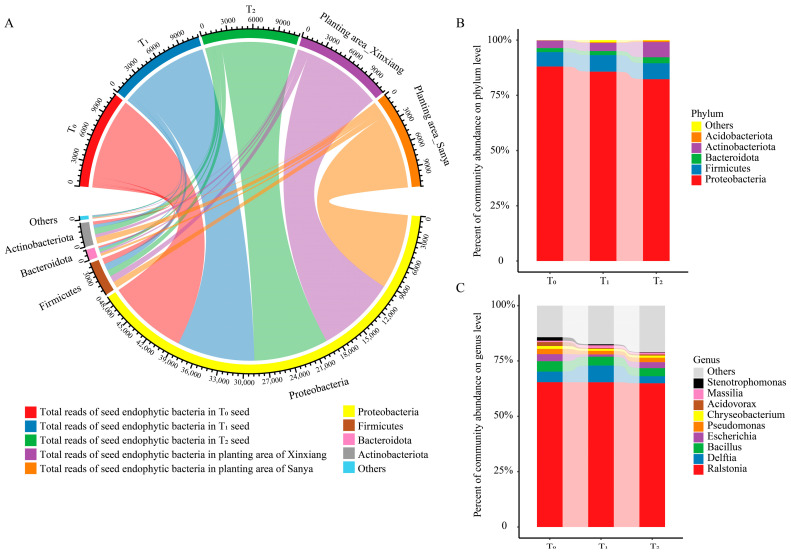
Community composition and taxa classification among three successive seed generations. (**A**) Chordal diagram of the bacterial community structure at the phylum level among three seed generations and two planting areas. Taxa with a proportion lower than 1% were summarized as Others. (**B**) Community composition at the phylum level in three seed generations. (**C**) Community composition at the genus level in three seed generations. T_0_, T_1_ and T_2_ represent three consecutive seed generations, respectively.

The dominant phylum was *Proteobacteria*, accounting for over 80% of total species, followed by *Firmicutes*, *Actinobacteriota* and *Bacteroidota* (Figure 2A,B). Moreover, there was no conspicuous difference among the three seed generations and the two planting areas at the phylum level (Figure 2A,B). *Ralstonia*, *Delftia*, *Bacillus*, *Escherichia*, *Pseudomonas*, *Chryseobacterium*, *Acidovorax*, *Massilia*, *Stenotrophomonas* and *Sphingomonas* were among the top 10 core genera, making up 79.66–85.58% of the total endophytic community (Figure 2C). *Bacillus* was affiliated with *Firmicutes* and *Chryseobacterium* was affiliated with *Bacteroidota*, while the other eight core genera belonged to *Proteobacteria*. Among them, *Ralstonia* was the dominant genus of maize seed endophytic bacteria, accounting for 65% of total OTUs.

**Figure 3:**
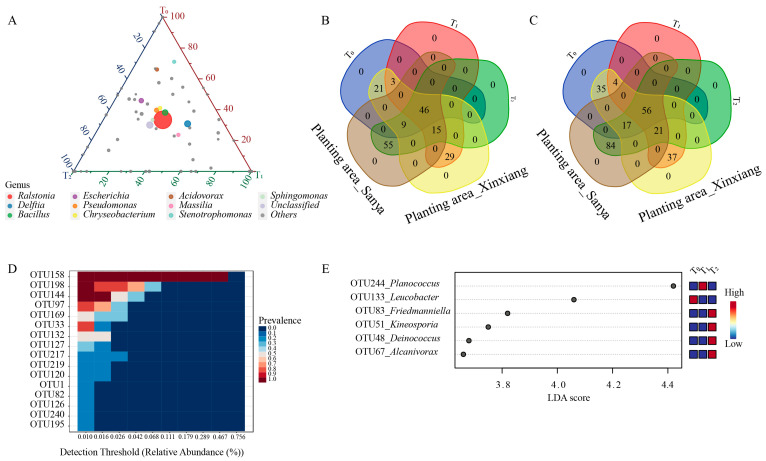
Distribution of seed endophytic bacteria among three seed generations. (**A**) Ternary plot of top 10 genera in three seed generations. (**B**) Venn diagram for shared and unique genera. (**C**) Venn diagram for shared and unique OTUs. (**D**) Relative abundance of core OTUs. (**E**) LEfSe analysis at OTU level among successive seed generations. Linear discriminant analysis (LDA) scores were obtained through LEfSe analysis. T_0_, T_1_ and T_2_ represent three consecutive seed generations, respectively.

Within the top 10 genera, *Ralstonia*, *Bacillus*, *Pseudomonas*, *Chryseobacterium* and *Sphingomonas* were nearly equally distributed among the three seed generations (Figure 2C, Figure 3A). *Acidovorax* and *Stenotrophomonas* were more abundant in the T_0_ generation, while *Delftia* and *Massilia* were more enriched in the T_1_ generation (Figure 3A). The abundance of *Escherichia* was similar between the T_0_ and T_1_ generations, but it was relatively lower in the T_2_ generation (Figure 3A). Through comparing the similarities and differences of the three successive seed generations and the two planting areas, 46 genera and 56 OTUs were found to exist in all three generations and in both planting areas, suggesting that these OTUs were conserved and could be transferred through vertical transmission (Figure 3B,C). Within the 56 OTUs, 16 of them were core OTUs with high abundance and prevalence, but only 10 OTUs were annotated at the species level (Figure 3D, Table 1). Moreover, there were 156 OTUs that were flexible among different seed generations and planting locations. The dominant OTUs were annotated to *Ralstonia solanacearum*, *Delftia tsuruhatensis* and *Bacillus velezensis* (Table 1). Moreover, there were 29 genera and 37 OTUs that were unique to Xinxiang, and 55 genera and 84 OTUs were unique to Sanya (Figure 3B,C). Through LEfSe analysis, six OTUs (OTU48, OTU51, OTU67, OTU83, OTU133, OTU244) showed prominent differences among three generations (Figure 3E). Among them, OTU244, belonging to *Planococcus*, was more enriched in the T_1_ generation, and OTU133, affiliated with *Leucobacter*, was more abundant in the T_0_ generation, while OTU48, OTU51, OTU67 and OTU83 were more enriched in T_2_ generation. The above results indicate that the conserved core OTUs could be transmitted vertically through seed generations, while some unique OTUs that exist within different seed generations could be transmitted horizontally for adaptation to external environments.

### 3.4. Three Seed Generations Shared Similar Functional Traits

**Figure 4:**
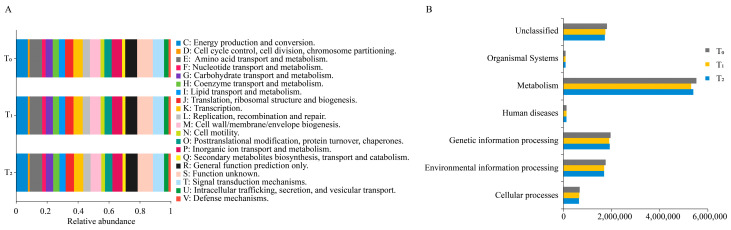
Metataxonomic function prediction of seed endophytic bacteria of three consecutive seed generations. (**A**) Function predictions were categorized based on COG classification. The abscissa represents the relative abundance of different functional categories based on the COG database, while the ordinate represents three consecutive seed generations. (**B**) Function predictions were categorized based on KEGG classification at level 1. The variation in the ordinate represents the different function items on the KEGG pathway level 1. T_0_, T_1_ and T_2_ represent three consecutive seed generations, respectively. The values in the abscissa represent the relative abundance of corresponding functions in the samples.

**Figure 5:**
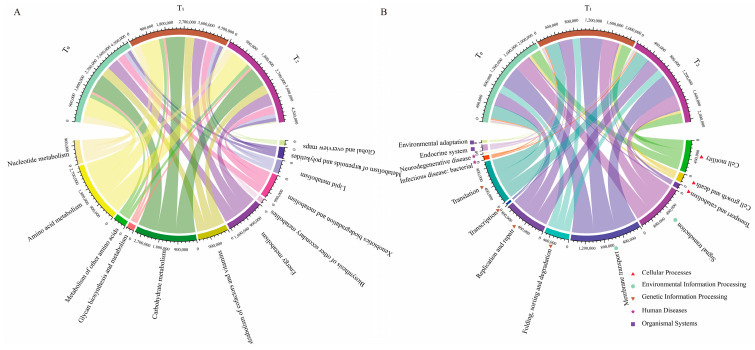
The major functions predicted for seed bacterial endophytes were enriched in metabolic functions. (**A**) Function predictions about metabolism were categorized based on KEGG classification at level 2. (**B**) Function predictions about genetic information processing, environmental information processing and cellular processes were categorized based on KEGG classification at level 2. T_0_, T_1_ and T_2_ represent three consecutive seed generations, respectively. The values represent the relative abundance of corresponding functions in the samples.

**Figure 6:**
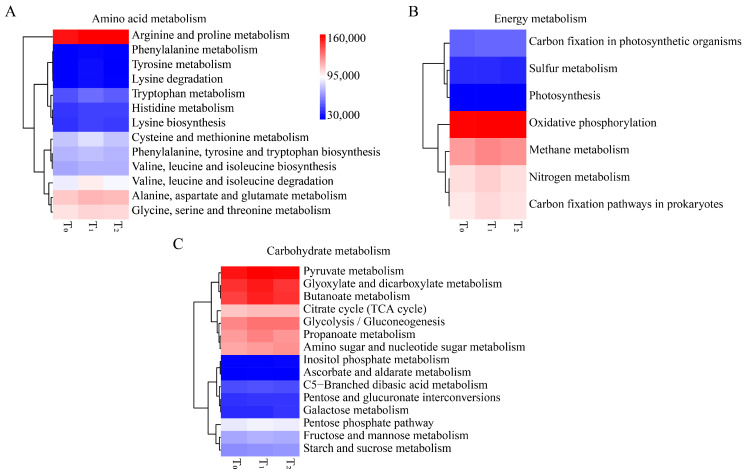
Functional prediction of maize seed bacterial endophytes that might involve amino acid metabolism, energy metabolism and carbohydrate metabolism. (**A**) Heatmap of relative abundance of amino acid metabolism. (**B**) Heatmap of relative abundance of carbohydrate metabolism. (**C**) Heatmap of relative abundance of energy metabolism. T_0_, T_1_ and T_2_ represent three consecutive seed generations, respectively. The values represent the relative abundance of corresponding functions in the samples, and the colors blue, white and red represent the values at low, middle and high levels, respectively.

To better understand the functions of seed endophytic bacteria, PICRUSt analysis based on the COG and KEGG databases was conducted. The results showed that the predicted functions for maize seed bacterial endophytes of three seed generations were highly conserved. Based on the COG database, the top enriched pathways were those involving amino acid transport and metabolism, energy production and conservation, signal transduction mechanisms, inorganic ion transport and metabolism, carbohydrate transport and metabolism, etc. (Figure 4A). In KEGG enrichment analysis, metabolic functions were the most important, followed by genetic information processing and environmental information processing (Figure 4B). Within the metabolic functions of KEGG, amino acid metabolism, carbohydrate metabolism and energy metabolism were dominant (Figure 5A). Moreover, replication and repair, membrane transport and cell motility were dominant in the pathways of genetic information processing, environmental information processing and cellular processes, respectively (Figure 5B). Furthermore, arginine and proline metabolism were dominant in amino acid metabolism, while oxidative phosphorylation and pyruvate metabolism were prominent in energy metabolism and carbohydrate metabolism, respectively (Figure 6).

## 4. Discussion

The maize seed is a suitable habitat for endophytic bacteria. Through coevolution, seeds and seed-borne endophytes have established a close symbiotic relationship and have benefited each other. In the present study, we found that maize seed endophytic bacteria community structure showed no significant changes among three generations in community α diversity. The above results are supported by research from Sanchez-Lopez et al. [28] in *Crotalaria pumila*, suggesting that the community diversity of seed bacterial endophytes was conserved to a certain extent across progeny transmission via seeds.

In nine monocot and eight dicot crop species, the top five phyla of seed endophytic bacteria were identified to be *Proteobacteria*, *Firmicutes*, *Cynaobacteria*, *Bacteroidota* and *Actinobacteriota* [29]. Our results showed that the dominant phylum of seed endophytic bacteria in maize was *Proteobacteria*, followed by *Firmicutes*, *Actinobacteriota* and *Bacteroidota*, which is accordant with previous reports in highland barley [30], *Crotalaria pumila* [28], *Solanum lycopersicum* [31], *Sophora davidii* [32], *Daucus carota* [33], *Elymus nutans* [34] and *Cistanche phelypaea* [35]. By analyzing endophytic bacterial community structure and diversity in melon seeds, another study identified 492 OTUs affiliated with 8 phyla, and *Proteobacteria* and *Firmicutes* were dominant [36]. Therefore, there is a high degree of conservation in the community structure of maize seed endophytic bacteria at the phylum level across seed generations and multiple crop species.

In maize, Liu et al. [14] reported that *Enterobacter*, *Shigella*, *Pseudomonas* and *Achromobacter* were the core genera of seed endophytes. From the seeds of 30 maize cultivars, 80 bacterial endophytes were isolated, and the dominant genera were *Bacillus* and *Staphylococcus* [37]. In the present study, the core genera of seed endophytes in maize were identified, including *Ralstonia*, *Delftia*, *Bacillus*, *Escherichia* and *Pseudomonas*. It is likely that the core genera in maize were highly variable, with only *Pseudomonas* and *Bacillus* being conserved. Zeng et al. [36] found that the core genera of melon seed endophytes were *Escherichia*, *Streptococcus*, *Bifidobacterium*, *Parabacteroides*, *Enterobacter* and *Roseburia*. *Bacillus*, *Massilia*, *Paenibacillus*, *Pantoea*, *Pseudomonas*, *Rhizobium* and *Sphingomonas* were highly abundant genera in *Daucus carota* [33], while the dominant endophytic bacteria of *Alpinia zerumbet* seeds were affiliated with *Pseudomonas*, *Kosakonia*, *Curtobacterium* and *Sphingobacterium* [38]. Through collecting seeds of *Elymus nutans* from four planting areas, one study found 10 core genera that were likely transmitted vertically across seed generations, including *Pseudomonas*, *Halomonas*, *Pantoea*, *Ochrobactrum*, *Massilia*, *Sphingomonas*, etc. [34]. Taken together, the core genera are different among different crop species and can be different even in the same crop species. Within them, *Pseudomonas*, *Bacillus*, *Massilia* and *Sphingomonas* exist ubiquitously, while *Ralstonia* and *Delftia* are rarely reported as core genera of seed endophytic bacteria.

In this study, we found that three consecutive seed generations shared 56 common OTUs. The dominant OTUs were *Ralstonia solanacearum*, followed by *Delftia tsuruhatensis*, *Bacillus velezensis*, *Shigella boydii*, *Acidovorax avenae*, *Pseudomonas mosselii*, *Stenotrophomonas maltophiliag* and *Staphylococcus hominis*. Wallace [27] reviewed publications about bacterial endophytes isolated from maize seeds up until 2022 and concluded that seven *Bacillus* strains (*B. altitudinis*, *B. amyloliquefaciens*, *B. aquimaris*, *B. cereus*, *pumilus*, *B. subtilis* and *B. velezensis*), *Paenibacillus dendritiformis*, *Staphylococcus arlettae*, *Burkholderia anthina* and *Pseudomonas aeruginosa* could be isolated from maize seeds. Pal et al. [39] found that endophytic bacterium *B. velezensis* within maize seeds could generate lipopeptides, which promoted seedling growth and activated ISR (induced systemic resistance) to enhance antifungal activity against the fungal pathogen *F. verticillioides*. Moreover, *Stenotrophomonas maltophilia* isolated from rice seeds showed an inhibitory effect on rice blast disease by suppressing the growth of *Magnaporthe grisea in vitro* when *S. maltophilia* was applied as biofertilizer in soil [40], while *Staphylococcus hominis* isolated from jute seeds also displayed antibacterial capability against *Staphylococcus aureus* [41]. In addition to *B. velezensis*, *Stenotrophomonas maltophilia* and *Staphylococcus hominis*, the functions of other core OTUs identified in this study remain to be characterized in the future.

**Figure 7:**
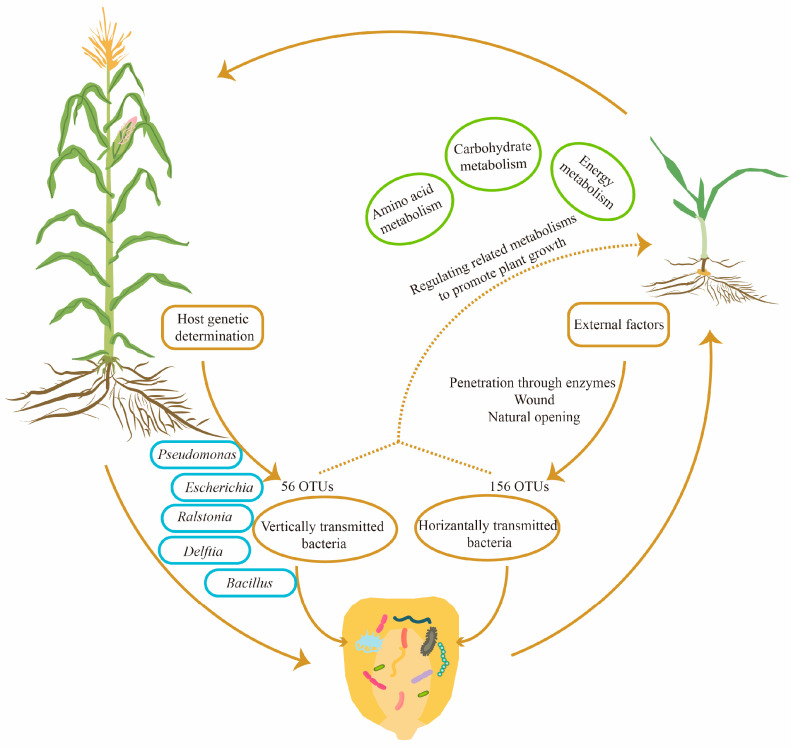
A diagram of the inheritance and potential functions of maize seed endophytic bacteria. According to the analysis of community structure among three consecutive seed generations, 56 OTUs were found to be conserved and transmitted vertically, whereas another 156 OTUs were changeable and transmitted horizontally from the external environment. Within the conserved 56 OTUs, 10 core OTUs were annotated at the species level. Based on COG and KEGG predictions, seed bacterial endophytes of maize might promote seedling growth by regulating amino acid metabolism, carbohydrate metabolism and energy metabolism.

Through investigating the COG and KEGG databases, we found that the functions of seed endophytic bacteria in maize were mainly involved in metabolic processes, particularly in regulating amino acid metabolism, carbohydrate metabolism and energy metabolism. The results were essentially consistent with findings in *Crotalaria pumila*, which also illustrated that the above three metabolic pathways were major functions of seed bacterial endophytes [28]. In tomato, KEGG functional prediction also proposed primary metabolic processes as the major role of seed bacterial community [42]. In *Cucumis melo*, the results indicated that the function of seed endophytic bacteria was enriched in pathways of protein synthesis, carbohydrate metabolism, substance transport and metabolite synthesis [36]. Taken together, diverse crop species are capable of exerting their functions using the conserved primary metabolic pathways of seed endophytic bacteria to facilitate seedling growth and development, as well as progeny propagation. The potential mechanisms by which seed endophytic bacteria promote crop growth are summarized in Figure 7.

## 5. Conclusions

The symbiotic relationship between seeds and their bacterial endophytes is of vital importance for plant growth and development. The current study focused on illustrating the inheritance and function prediction of maize seed bacterial endophytes. Fifty-six OTUs were conserved across three successive seed generations. Within them, *Ralstonia*, *Bacillus*, *Pseudomonas*, *Chryseobacterium* and *Sphingomonas* are core genera of maize seed bacterial endophytes. Host plants could maintain these conserved core OTUs across progeny transmission, which may benefit seedling growth and survival in the face of environmental stresses or pathogen attacks through modulating the metabolic process.

## Figures and Tables

**Table 1:** Annotation information of core OTUs at species level.

OTU	Reference Species	Abundance
OTU217	*Acidovorax avenae*	0.75%
OTU169	*Bacillus velezensis*	1.31%
OTU198	*Delftia tsuruhatensis*	4.32%
OTU120	*Pseudomonas mosselii*	0.56%
OTU158	*Ralstonia solanacearum*	54.31%
OTU82	*Sanguibacte rinulinus*	0.21%
OTU132	*Shigella boydii*	1.12%
OTU32	*Sphingomonas paucimobilis*	0.23%
OTU1	*Staphylococcus hominis*	0.31%
OTU219	*Stenotrophomonas maltophiliag*	0.49%

## Data Availability

Dataset available on request from the authors. The raw data supporting the conclusions of this article will be made available by the authors on request via dataset-file. The 16S rDNA sequences from the obtained isolates were submitted to the NCBI database with the URL of “https://www.ncbi.nlm.nih.gov/bioproject/1188770”.
